# Detection and quantification of poliovirus infection using FTIR spectroscopy and cell culture

**DOI:** 10.1186/1754-1611-5-16

**Published:** 2011-12-05

**Authors:** Felipe T Lee-Montiel, Kelly A Reynolds, Mark R Riley

**Affiliations:** 1 Agricultural and Biosystems Engineering, University of Arizona, Tucson, Arizona, USA 85721; 2 Mel and Enid Zuckerman College of Public Health, University of Arizona, Tucson, Arizona, USA 85724

**Keywords:** Enterovirus, Fourier Transform Infrared (FTIR) spectroscopy, zinc selenide (ZnSe), mid-infrared, partial least squares, cell culture, buffalo green monkey kidney (BGMK) cells, virus detection, poliovirus (PV1)

## Abstract

**Background:**

In a globalized word, prevention of infectious diseases is a major challenge. Rapid detection of viable virus particles in water and other environmental samples is essential to public health risk assessment, homeland security and environmental protection. Current virus detection methods, especially assessing viral infectivity, are complex and time-consuming, making point-of-care detection a challenge. Faster, more sensitive, highly specific methods are needed to quantify potentially hazardous viral pathogens and to determine if suspected materials contain viable viral particles. Fourier transform infrared (FTIR) spectroscopy combined with cellular-based sensing, may offer a precise way to detect specific viruses. This approach utilizes infrared light to monitor changes in molecular components of cells by tracking changes in absorbance patterns produced following virus infection. In this work poliovirus (PV1) was used to evaluate the utility of FTIR spectroscopy with cell culture for rapid detection of infective virus particles.

**Results:**

Buffalo green monkey kidney (BGMK) cells infected with different virus titers were studied at 1 - 12 hours post-infection (h.p.i.). A partial least squares (PLS) regression method was used to analyze and model cellular responses to different infection titers and times post-infection. The model performs best at 8 h.p.i., resulting in an estimated root mean square error of cross validation (RMSECV) of 17 plaque forming units (PFU)/ml when using low titers of infection of 10 and 100 PFU/ml. Higher titers, from 10^3 ^to 10^6 ^PFU/ml, could also be reliably detected.

**Conclusions:**

This approach to poliovirus detection and quantification using FTIR spectroscopy and cell culture could potentially be extended to compare biochemical cell responses to infection with different viruses. This virus detection method could feasibly be adapted to an automated scheme for use in areas such as water safety monitoring and medical diagnostics.

## Background

Increased population density and movement of people around the globe have generated a rise in the number of outbreaks of infectious diseases and led to the emergence of new infectious diseases [1]. Worldwide, 3.575 million people die each year from water-related diseases [2]. The water and sanitation crises claim more lives through disease than any warfare [2]. A key step in the prevention of outbreaks of communicable diseases is the early detection of virulent particles [3]. Rapid detection of active viral pathogens is of central importance for public health risk assessment and environmental protection. Waterborne viruses are particularly important for public safety monitoring due to their environmental stability and low infectious dose; a single virion is sufficient to initiate illness in previously unexposed, healthy adults [4].

Enteroviruses (family Picornaviridae) are a genus of waterborne viruses that infect humans and other mammals. They are a health problem worldwide, leading to 10 to 15 million cases of symptomatic infection in humans annually in the United States alone [5]. Enteroviruses are single, positive-strand RNA viruses that include polioviruses, Coxsackieviruses and echoviruses, among others. Some enteric virus groups have emerged as waterborne pathogens because of their high levels of resistance to current water treatment processes, which include ultraviolet light inactivation and heat inactivation [6,7]. Poliovirus was used here as a model virus because a large body of research data exists on the physical, chemical and biological properties of the virus, vaccination is available, and its ease of cell culturing [8-10]. In addition, poliovirus remains endemic in four countries. During 2002 the rejection of polio immunization led to a worrying resurgence of polio in some areas of Nigeria, followed by re-infection in 21 other countries; resurgence of the disease was also observed in India. Auxiliary vaccination actions were restarted and by 2007 most re-infected countries had become polio-free again. The goal of global polio eradication was re-set to 2010, but concerns continue to be expressed about the progress of this eradication program [11].

Current methods for enterovirus detection use mammalian cell culture and require complex analyses (visible monolayer cytopathic effects) that require several days of laboratory time [12]. Polymerase chain reaction (PCR) methods for the detection of viruses have been developed, offering specificity, speed and cost advantages over cell culture methods [13,14]. PCR methods alone do not, however, differentiate between the presence of physical (inactive) virus particles and viable (active) virus particles [6,15]. The major disadvantage of most current methods of virus detection is the inability to provide information about whether a viral particle can start an infection or not. Faster methods with increased sensitivity and specificity are needed to quantify active viral pathogens from medical and environmental samples.

Virus-infected individuals can excrete over 1 billion (10^9^) viruses/g of feces. Some enteric viruses can also be excreted in urine from infected individuals. The presence of these viruses in a human population is variable and reflects current epidemic and endemic conditions [16]. In general, the level of infectious enteric virions in sewage ranges from 100 to 10,000 infectious units/L [17-23]. In contaminated surface water, levels of 1-100 infectious enteric virions/L are common. In less polluted surface water, their numbers are closer to 1-10/100 L. Groundwater sources have been shown to have between 0 and 200 infectious enteric virions/100 L, depending on the level of contamination; however, most contaminated groundwater systems are thought to have very low levels (< 2/100 L) [24]. These concentrations were generally obtained through targeted studies, since water and wastewater sources are not routinely monitored for enteric viruses. These measurements are typically performed after an extensive concentrating step which reduces sample volume by 100 to 1000-fold before virus detection is performed.

FTIR spectroscopy is a noninvasive measurement method that has previously been applied for identifying various biological components of cells by detecting vibrations of molecules leading to spectral patterns [12,25]. It can be used as part of a sensitive method for the detection of specific cellular molecular changes [12,26-30]. Quantitative infrared absorption methods such as FTIR spectroscopy differ from ultraviolet/visible molecular spectroscopic methods because of the greater information content of the spectra. FTIR spectroscopy has been applied in medicine, particularly to study the process of herpes virus infection [31] and in the diagnosis of cancers and other disorders [32-35]. In addition, FTIR spectroscopy has been used for the quantification of blood serum components such as glucose, protein, cholesterol and urea [36].

Cell based sensors detect changes in the physiological state of cells following exposure to an environmental stimulus. Changes in cell state can provide information about the stimulus; for example, cells have been used to sense toxins in water samples [37,38]. Cell based sensors have recently been combine with spectroscopy and applied to viral detection [39,40]. Cantera et al. [41] used an optical system that involved the use of molecular beacons as a way to detect infective virus particles, resulting in a detection limit of 1 PFU. Using live cells to assist in identifying and quantifying viruses in samples helps to bring the detection closer to an *in vivo *setting, allowing the natural and complex interactions between cell and virus to be part of the experimental setup.

**Figure 1:**
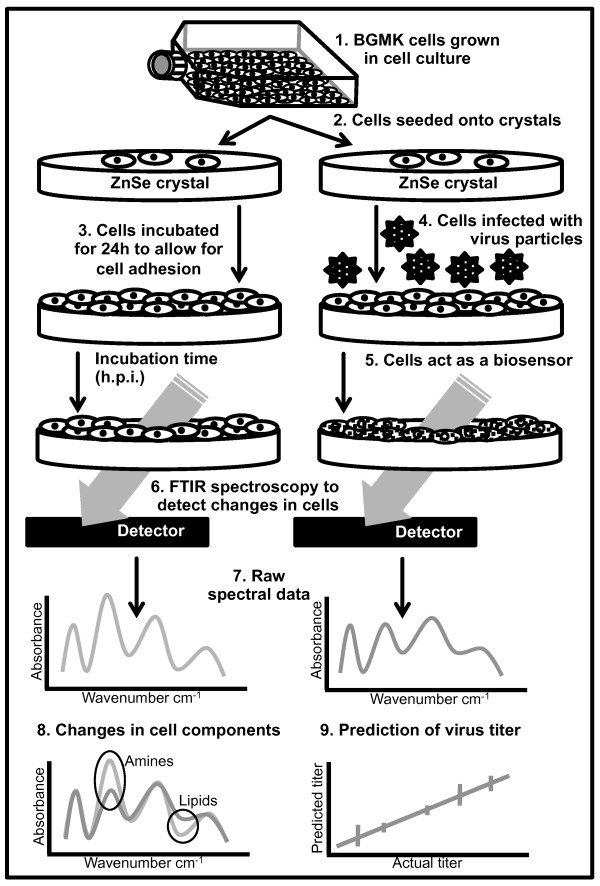
**Schematic representation of viral detection method using cell culture and FTIR spectroscopy (not to scale)**.

Here we present the development of a novel strategy for virus detection using a combination of FTIR spectroscopy and live BGMK cells (Figure 1). Specific absorbance patterns are monitored following changes in cell components (such as lipids, proteins, nucleic acids and sugars) subsequent to the virus infection, effectively using the cells as biosensors [42,43].

## Results

### Microscopy of cell structure on crystal

**Figure 2:**
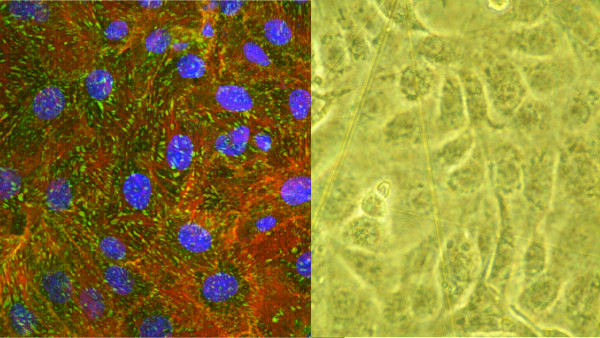
**Microscopy of BGMK cells attached on ZnSe crystal (actin filaments, vinculin and nuclei) and bright field image of BGMK cells**. a) Confocal image of BGMK cells adhered to a ZnSe crystal. Actin (red), Vinculin (green) and cell nuclei (blue) are shown. b) Bright microscopy image of BGMK cells adhered to a ZnSe crystal.

The way in which cells attach in monolayer culture depends on the cell type and the characteristics of the surface. BGMK cells were confirmed to be biocompatible with the ZnSe crystals for transmission measurements. The organization of the actin cytoskeleton exhibited a good distribution throughout the cell volume with many focal adhesion points that resulted in a spread phenotype on the stiff surface, indicating actin assembly by healthy cells. The cell architecture of the BGMK cells attached to the ZnSe crystal can be seen in Figure 2a. Actin, vinculin and cell nuclei were stained to study cell adhesion of BGMK cells on the ZnSe crystal. Figure 2b shows the cells under bright field microscopy, where they can be seen elongated and spread out over the crystal surface.

### Optimal time for virus detection

**Figure 3:**
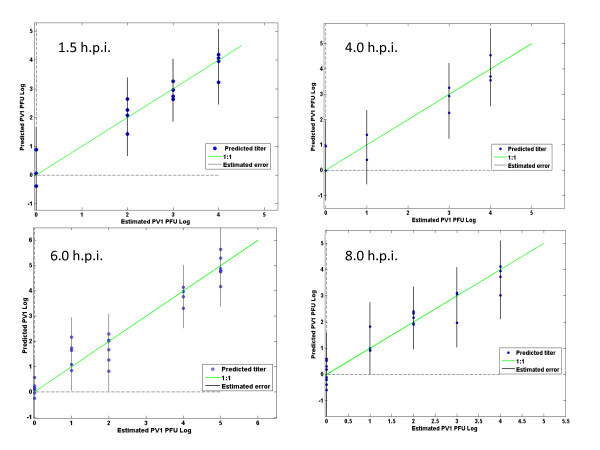
**Poliovirus prediction models comparing the estimated PFU and predicted PFU at 1.5 - 8 h.p.i**. Each point represent the predicted number of virus by the model for each sample exposed to different viral titers, × axis represent the estimated number of virus used.The green lines indicate a 1:1 regression model.

BGMK cells were infected with poliovirus PV1 at different multiplicities of infection (m.o.i.) of 10 PFU (0 - 10^6 ^PFU/ml) and studied at 1, 1.5, 2, 4, 5, 6, 8 and 12 h.p.i. Virus infection regression models were developed to correlate changes in spectral features with time of infection. Changes in the spectra varied depending on the progress of the viral infection, with biochemical alterations appearing in poliovirus infected cells within 2 h.p.i. Example regression models for 1.5, 4, 6 and 8 h.p.i. are shown in Figure 3 and a summary of the regression model parameters are given in Table 1. This table shows the comparison between the error of calibration (RMSEC) and the root mean square error of crossvalidation, RMSECV, which is a mesure of a model's ability to predict samples that were not used to build the model (leave-one-out-crossvalidation). RMSECV was analyzed to determine the optimum number of latent variables (LVs) to include in the PLS model. The number of LVs of a PLS model is usually optimized by performing a cross-validation and minimizing the corresponding RMSECV. The RMSECV decreases with the inclusion of each additional initial factor, reaching a minimum value with a certain number of latent variables. The best choice of number of latent variables is also supported by the variance capture in Y. The goal is to identify a subset of the measured variables that gives the lowest RMSECV, which is the most useful and accurate regression model. General calibration procedure consists in the selection of the pretreatment (preprocessing of the spectra), wavelength intervals, and number of latent variables to be driven by minimizing the RMSECV [44]. An infection time of 8 h was selected for subsequent experiments based on these results and on the reported viral replication time [44]. For more information on the spectra of 8 h and 12 h uninfected control cells see Additional File 1 and Additional File 2 respectively.

**Table 1:** Comparison of poliovirus prediction models using different infrared regions and virus titers

Method	IR region used	h.p.i	Virus titers (PFU)	Latent variables	R^2^	RMSEC	RMSECV
**Initial**	650 - 1650 cm^-1^	1.5	0, 10^2 ^- 10^4^	4	0.910	0.4225	0.7093
	
	650 - 1650 cm^-1^	4	0, 10^1^, 10^3^, 10^4^	3	0.891	0.5225	0.8900
	
	650 - 1650 cm^-1^	6	0, 10^1^, 10^2^, 10^4^	6	0.868	0.4914	0.8800
	
	650 - 1650 cm^-1^	8	0 - 10^4^	7	0.911	0.4290	0.8471

**Revised**	650 - 3600 cm^-1^	8	0 - 10^4 ^	7	0.819	0.6480	0.9187
	
	650 - 1650 cm^-1^	8	0 - 10^3 ^	7	0.917	0.3298	0.5726
	
	iPLS 9 regions	8	0 - 10^3 ^	7	0.903	0.3577	0.5581
	
	iPLS 9 regions	8	0, 10^2 ^- 10^4^	7	0.944	0.4019	0.6628
	
	iPLS 9 regions	8	0 - 10^2^	7	0.964	0.1640	0.3163
	
	650-1650 cm^-1^	12	0 - 10^3^	7	0.716	0.5963	1.2866

Table shows root mean square error of calibration (RMSEC), root mean square error of cross validation (RMSECV) and correlation coefficient for each model.

### Viral infection at 8 h.p.i

**Figure 4:**
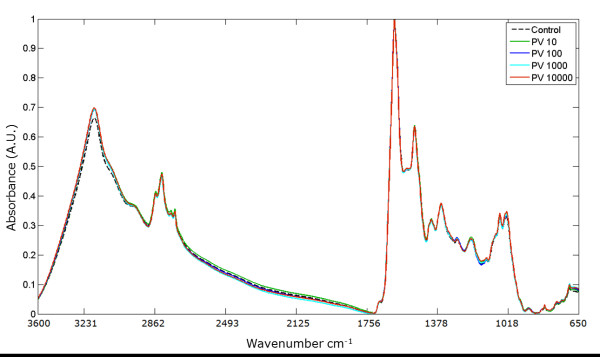
**FTIR spectra showing changes in absorbance when cells are infected with poliovirus**. Spectra in the wavelength region of 650 - 3600 cm**^-1 ^**show the absorbance of BGMK cells infected with different PV1 titers 10**^1 ^**- 10**^4 ^**PFU/ml at 8 h.p.i. Uninfected cells served as a control. Nine regions were chosen by the PLS model as the most informative for detecting changes in cell components following virus infection. The different colors represent the mean spectra of the samples.

Spectra of cells at 8 h.p.i infected with different m.o.i. of PV1 are shown in Figure 4. Interval Partial Least Square (iPLS) was performed to determine the optimal regions of the spectra used in the virus detection model, an interval size of 10 cm^-1 ^and a maximum of 8 latent variables were chosen. These 9 areas of the spectra correspond to the following wavenumbers: 660.90 - 667.26, 806.11 - 823.46, 844.68 - 862.03, 979.67 - 997.03, 1095.38 - 1112.74, 1191.80 - 1209.16, 1230.37 - 1267.02 and 1326.80 - 1344.16 cm^-1^.

Changes in absorbance can be correlated with the development of poliovirus infection. The region between 600 - 900 cm^-1 ^corresponds to C2' endo/anti (B-form helix) conformation, DNA and RNA molecules. 1000 to 1300 cm^-1 ^relates to symmetric stretching mode of dianionic phosphate monoester in phosphorylated proteins and left handed helix DNA (Z form) [45].

**Figure 5:**
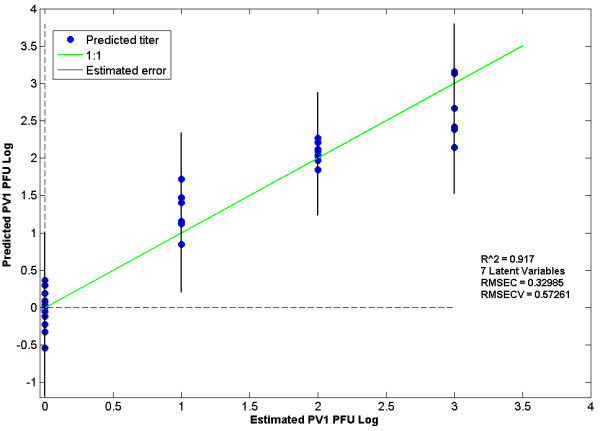
**Regression analysis for cells infected with PV1 at 8 h.p.i**. This model uses 7 latent variables. The regression uses a log scale and 0 - 10**^3 ^**PFU/ml in the 650 - 1600 cm**^-1 ^**wavenumber region.

Figure 5 shows a regression model of poliovirus at 8 h.p.i. that correlates changes in absorbance spectra with virus infection titer. This model has a root mean square error of cross validation (RMSECV) of 0.57 log. Similar results were achieved using the whole spectral region or the nine regions selected by iPLS (data not shown). This method can detect a viral titer of 10^1 ^- 10^2 ^PFU/ml with a RMSECV of 17 PFU/ml; at higher titers, 10^2 ^- 10^4 ^PFU/ml, a RMSECV of 2009 PFU/ml for 8 h.p.i. was achieved.

### Effect of virus titer on characteristic spectra peak height at 8 h.p.i

**Figure 6:**
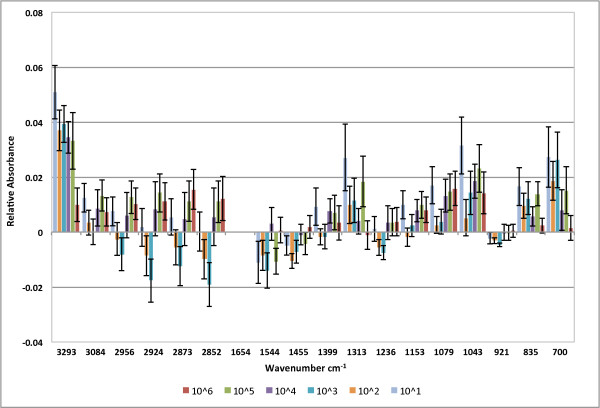
**Average peak absorbance values compared to uninfected control for different virus titers at 8 h.p.i**. Error bars show standard error. Note that 1654 cm^-1 ^was used to normalize the data and therefore shows no change.

The characteristic peaks of the BGMK cell line showed changes in relative absorbance following infection with different viral titers. These peaks correspond to different biomolecules. A graphical representation of the absorbance values of the eighteen characteristic peaks of each data set for the different infection titers (10^6^, 10^5^, 10^4^, 10^3^, 10^2 ^and 10^1 ^PFU/ml) for the 8 h.p.i. time point is given in Figure 6. A summary of the change in peak height upon virus infection and the corresponding biomolecules represented at the peak wavenumbers are given in Table 2.

**Table 2:** Observed changes in relative peak absorbance values with corresponding biomolecules

Peak wavenumber (cm^-1^)	Corresponding biomolecules	Observed changes in cell absorbance following PV1 infection relative to uninfected control cells
**700**	(Broad) cis-C-H out-of-plane bend	General increase

**835 - 840**	C2' endo/anti (B-form helix) conformation and left handed helix DNA (Z form)	Gradual increase corresponding to increased PV1 titer

**900 - 1350**	Phosphodiester stretching bands (from absorbance due to collagen and glycogen)	Decrease; trend is less clear at higher PV1 titers

**1040 - 100**	Symmetric stretching vibration PO_2 _due to RNA and DNA	General increase

**1079/80**	Symmetric phosphate stretching modes or ν(PO2 −) symmetric. Phosphate stretching modes originate from the phosphodiester groups in nucleic acids.	Sharp increase in BGMK cells infected with 10^1 ^PFU/ml titer followed by a decrease in the 10^2 ^PFU/ml infected cells then a gradual height incremental with the higher titers.

**1153**	Stretching vibrations of hydrogen bonding C-OH groups	Virus titer 10^2 ^PFU/ml shows a decrease

**1236/7**	Amide III, stretching PO_2_−asymmetric (phosphate I)	Decrease in the peak height for the cells infected with 10^2 ^and 10^3 ^PFU/ml; higher titers shown an increase

**1312-1317**	Collagen related, amide III band components of proteins	General increase

**1455/6**	Asymmetric CH_3 _bending modes of the methyl groups of proteins	General decrease

**2852, 2873, 2924/5, 2956**	Lipids region (CH_2 _symmetric, symmetric stretching vibration of CH_3 _of acyl chains, stretching C-H and asymmetric stretching vibration of CH_3 _of acyl chains respectively)	Decrease for infection titers 10^2 ^- 10^3 ^PFU/ml

**3084**	Stretching N-H symmetric	General increase

**3293**	OH stretching (associated)	General increase; increase gradually less from 10^1 ^- 10^6 ^PFU/ml titers

Some trends are evident for the change in peak heights at different titers. For example, lower virus concentrations (10^2 ^and 10^3 ^PFU/ml) showed negative values relative to controls at 1399 - 2956 cm^-1^, indicating a decrease in the amount of a specific biochemical component. In contrast, the mean absorbance of the cells with higher virus titers (10^4^, 10^5 ^and 10^6 ^PFU/ml) only showed average increases in absorbance across all peaks relative to uninfected controls.

The most significant differences were found for the peak at 3293 cm^-1^, assigned to OH stretching (One-way ANOVA, d.f. 6, F = 7.63, p < 0.0001), for which the 10^6 ^PFU/ml sample had significantly lower absorbance than the 10^1 ^PFU/ml sample (Tukey-Kramer HSD, α = 0.05). The 10^6 ^PFU/ml sample and uninfected control had significantly lower absorbance than the 10^1 ^- 10^5 ^PFU/ml samples at this wavenumber (Tukey-Kramer HSD, α = 0.05). A significant difference was found for the peak at 1043 cm^-1 ^assigned to glycogen (One-way ANOVA, d.f. 6, F = 2.47, p = 0.032) [46,47] for which the absorbance of the 10^1 ^PFU/ml sample was significantly higher than the absorbance of the uninfected control (Tukey-Kramer HSD, α = 0.05). Alterations in glycogen have been described in neurons of monkeys infected with poliovirus [48].

The mean absorbance values for the different titers at the 700 cm^-1 ^(ANOVA, d.f. 6, F = 2.37, p = 0.0383) and 835 cm^-1 ^(ANOVA, d.f. 6, F = 2.36, p = 0.0387) peaks also formed significantly separate groups. The absorbance for the 1079 cm^-1 ^(ANOVA, d.f. 6, F = 2.08, p = 0.0659), 1313 cm^-1 ^(ANOVA, d.f. 6, F = 1.99, p = 0.0781), 2852 cm^-1 ^(ANOVA, d.f. 6, F = 1.89, p = 0.0942), 2924 cm^-1 ^(ANOVA, d.f. 6, F = 2.16, p = 0.0575) titers showed consistent trends.

### CPE analysis

The error associated with a standard CPE analysis was compared to the error of the new FTIR spectroscopy with cell culture method. The results of this assay are shown in Table 3. Standard error was calculated from duplicate experiments except where there were too many PFUs to count accurately. The average standard error was 12.0% of the mean estimated number of PFU/ml. For comparison, the error of cross validation was ~17% of the mean across titers using the FTIR with cell culture method. Note that these calculations assume a perfect serial dilution to obtain the starting known viral titers.

**Table 3:** CPE Analysis for Poliovirus at a virus titer of 10^7 ^PFU/ml.

Sample of Known Titer (PFU/ml)	Dilution Factor	Estimated PFU/ml	**S.E**.
**1000000**	-6	7000000	2000000

**100000**	-5	550000	50000

**100000**	-4	380000	40000

**10000**	-4	35000	5000

**10000**	-3	39500	2500

**1000**	-3	4000	0

**1000**	-2	3300	100

**10**	-1	5	5

## Discussion

Cells infected with poliovirus PV1 showed consistent alterations in their infrared spectral features. The broad, undulating features observed relatively weakly in the spectra of entire cells has been previously attributed to Mie scattering of the cellular nuclei [49,50]. The overall variations in intensity in the initial method are most likely due to variations in the thickness of the cell, as well as the nucleus/cytoplasm (N/C) ratio.

Multiple chemometric models were developed using a variety of pre-processing approaches, different spectral regions and different latent variables. This method could detect a viral titer of 10^1 ^- 10^2 ^PFU/ml with a RMSECV of 17 PFU/ml in cells at 8 h.p.i. At higher titers the accuracy decreased slightly, for example at 10^2 ^- 10^4 ^PFU/ml, a RMSECV of 2009 PFU/ml for 8 h.p.i. was achieved. Changes in cell spectral features become apparent initially at 2 h.p.i, but detection accuracy is best at 8 h.p.i.

Changes in cell absorbance spectra were correlated with specific cell components to better understand the progress of the poliovirus infection. The most significant changes were associated with an increase in the peak assigned to OH stretching, particularly at low virus titers. Another significant change associated with infection was an increase in the peak assigned to symmetric stretching vibration of PO_2 _due to RNA and DNA, which was most evident in the 10^1 ^PFU/ml virus titer. Broad cis-C-H out of plane bend and center handed helix of DNA also showed significant changes associated with PV1 infection.

To obtain the high level of sensitivity required substantial method development steps. Initial experiments resulted in models with a high level of error. In the initial approach cells were grown in a culture flask, trypsin was used to detach the cells, they were centrifuged and transferred to the ZnSe crystals for transmission measurements. Growing cells directly on the thin ZnSe crystals used a greatly simplified protocol, removing many steps where error and sample handling variation could occur. The direct cultivation method produced cleaner, more stable spectral patterns and had a higher reproducibility compared to the initial method.

This virus detection method was consistently more accurate for lower virus titers. This could be because the lower titers cause less variation in the viral infection process of adsorption and penetration inside of each individual cell. For example, at low titers the virus particles may be invading the cells in synchrony, causing more equal changes in the cell components among cells. With many virus particles present there might be more than one virus infecting each cell, meaning that the stage of infection is not synchronized among cells. Cantera et al. [41,51] have reported greater success for detection of lower virus titers of poliovirus after 12 h.p.i. It may be that for lower virus infection it is necessary to wait longer until the cells reach some steady infection state so that the changes in cell components are saturated, causing the overall signal pattern to stabilize.

**Figure 7:**
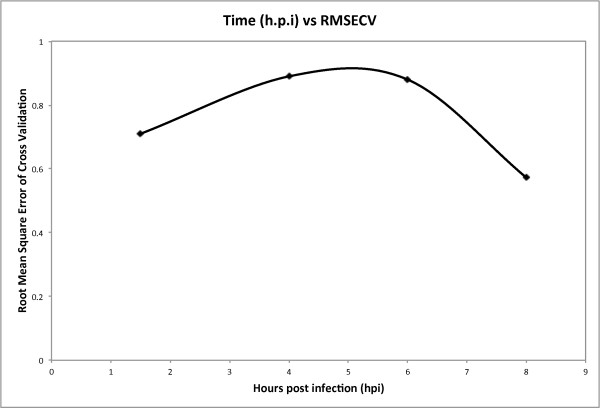
**Hours Post-Infection vs. Root Mean Square Error of Cross Validation**. Graph shows the corresponding RMSECV for the experiments using 1.5, 4, 6 and 8 h.p.i. The lowest RMSECV value was achieved using an infection time of 8 h.p.i.,which corresponds to the best prediction model.

An infection period of eight hours had the lowest RMSECV of the times tested here (Table 1, Figure 7). Poliovirus replicates and lyses the cell in approximately 8 h [44] and a previous study using molecular beacons also found this time period to be the best for viral quantification [41]. Eight hours of infection was found to be the best for detection of poliovirus with a method based on engineered BGMK cells expressing fluorescent proteins undergoing fluorescence resonance energy transfer (FRET) [51] and [52]. Perhaps this is the time point when the most dramatic changes in cell components are occurring [51], meaning that the eight hour time point in our protocol may be just prior to virus release from infected cells, producing a better prediction model of the infection (Figure 7). Note that our method does not require any modification to the sacrificial cells and as such can have the recognition element readily changed.

A CPE assay is the standard method for enterovirus detection and requires three to fourteen days to perform depending on the virus type. The poliovirus assay takes three to ten days during which the presence of a virus is marked by the death of animal cells in culture [3]. The cells are grown in a monolayer in a semi-solid media such that new virus particles infect surrounding cells. Cells killed by the virus form a plaque and the remaining living cells are stained with a dye. Another method, integrated cell culture-PCR (ICC-PCR), is a faster molecular detection method that can identify low levels of viable virus [3]. A sample is applied to cell culture and then molecular methods are used to detect replicating virus in the cell culture. This method takes 1 to 3 days from infecting the cells, depending on the replication time of the virus [13].

The cell culture-FTIR process has a distinct advantage over standard PCR methods since it provides information on viral infectivity and is inherently a multiplexed method; it does not however provide information on the specific type of viral infection. We envision this approach as being on the front end of a comprehensive screening process upstream of a more time consuming but more specific method such as cell culture-PCR to later determine the identity of the virus only for samples that indicate the presence of such an infective component.

## Conclusions

Here we present a fast new method for the detection of low titers of viable poliovirus using mammalian cells as a biosensor combine with FTIR spectroscopy to detect changes in cell components. Virus titers from 10^1 ^- 10^6 ^PFU/ml were assayed over a range of infection times from 1 h.p.i. - 12 h.p.i., with detection most accurate at low titers from 10^1 ^-10^4 ^PFU/ml. Prediction models of infected cells were best detected as early as 6 h.p.i., and had the most accurate prediction of virus titer at 8 h.p.i. The model gave a RMSECV of 17 viral particles. The major changes in cell components following virus infection were an increase in the peak 3293 cm^-1 ^and negative trend in the absorbance for lipids region (3200 - 2800 cm^-1^) in the poliovirus infected cells with 10^2 ^- 10^3 ^PFU/ml.

The cell culture method is still considered the standard for viral diagnosis as it has the advantages of detecting infectious viral particles and the ability to achieve low detection limits [6]. Combining the cell culture method with FTIR spectroscopy, we can enhance the cell culture method with the increased speed of FTIR spectroscopy. This new method has the potential to be extended for the detection of other viruses and adapted into a portable, automated system for detection of viruses from environmental samples.

## Methods

### Protocol development

An overview of the method is presented in Figure 1. Experiments were performed in a biosafety cabinet under laminar flow to maintain aseptic conditions. Our first experiments involved growing cells on the plastic surface of 24-well plates, removing the cells with an enzymatic treatment, pelleting the cells by centrifugation and then transferring them to ZnSe crystals for transmission measurements, allowing them to dry on the crystal to scan using FTIR spectroscopy. Spectra were collected in a transmission mode with light passing through both the cells and the underlying ZnSe crystal. Several changes were made to the initial method to optimize both the laboratory steps and the spectra results by reducing the room for variation between experimental replicates. We present both methods for comparison.

### Cell culture

BGMK cells were selected due to their high sensitivity to enteroviruses and previous use [53]. Cells were grown in T25 flasks to a confluent monolayer in Dulbecco's Modified Eagle Medium (DMEM) with 584 mg/L L-glutamine (Cellgro) containing 0.1% NaHCO_3_, 10,000 units/ml penicillin, 10,000 μg/ml streptomycin (HyClone) and 10% (vol/vol) fetal bovine serum (FBS; HyClone) and buffered with 12 mM HEPES. Reagents were from Sigma Chemical Co. unless otherwise noted. Cells were cultured at 37°C in a humidifying incubator with 5% carbon dioxide and passaged when confluent, approximately every two days. Cells were detached from the growth flask with 2.5% EDTA-trypsin, centrifuged at 18 g for 8 minutes, resuspended in media and transferred into a flask of fresh media.

### Initial method

T25 flasks with 95% confluent cell monolayers were trypsinized, resuspended in media and seeded in the eight central wells of a 24-well plate. The wells were individually seeded with 125,000 cells in 1 ml of media. Cells were grown to 100% confluence by incubating for 24 h at 37°C and 5% CO_2_. 0.5 ml of media was removed from the well and replaced with 0.5 ml of virus. A minimum of two control samples per experiment were inoculated with virus-free media using the same incubation time for infected and uninfected cells. Following viral exposure, cells were detached with 70 µL of trypsin per well and pelleted by centrifugation at 18 × g for 8 minutes. The cells were washed with 1 ml of 0.9% NaCl to remove any residual cell media then resuspended in 20 μL of 0.9% NaCl saline solution.

### Revised method

Several changes to the initial method were made to reduce the room for variation between experimental replicates, including growing the cells directly on the ZnSe crystals for transmission measurements. Individual ZnSe crystals were located in the eight central wells of a 24-well plate and seeded with 150,000 cells with a final volume of 1 ml DMEM media per well in a 24-well plate and incubated for 24 h to allow cell attachment to the crystal surface. After the 24 h incubation time, 0.5 ml of media was removed from the well and replaced with 0.5 ml of the virus titer. A minimum of two control samples per experiment were inoculated with virus-free media and incubated for the same period of time.

Following the virus infection, ZnSe crystals were removed from the aqueous medium allowing them to dry at room temperature for a minimum of 8 h. Spectra were collected in a transmission mode with light passing through both the cells and the underlying ZnSe crystal. An estimated 100,000 cells were scanned per ZnSe crystal. ZnSe crystals were washed with soap, pure acetone and sterilized in 70% EtOH between uses. Experiments with different infection times were performed with the initial method. The 8 h.p.i. and 12 h.p.i. experiments were subsequently performed with the revised method.

### Virus titers and infection times

Multiple serial dilutions were made from the initial stock concentration of 10^7 ^PFU/ml of purified vaccine strain PV1 (LSc-2ab) using different time of infection experiments. Previous studies observed molecular and morphological changes in poliovirus infected cells within 2 h.p.i. [44]. This infection time was therefore used as a starting point for identification of the optimum incubation time. BGMK cells were infected with PV1 at m.o.i. of 10 PFU (0 - 10^6 ^PFU/ml) and studied at 1, 1.5, 2, 4, 5, 6, 8 and 12 h.p.i. Different viral titers were made using DMEM with 10% NCS.

### FTIR spectroscopy

Infrared spectra of healthy cells and cells post-infection were collected in transmission mode on a ThermoNicolet Magna 560 FTIR spectrometer equipped with a liquid nitrogen-cooled MCT-A detector, KBr beamsplitter and infrared light source. The spectral collection parameters used were 128 co-added scans and a spectral resolution of 4 cm^-1 ^with a 20% aperture opening, performed using OMNIC 7.3 (Thermo Electron Corporation).

### Preprocessing of spectra

Preprocessing was performed to reduce noise and other interferences in the data. This included computation of the first derivative of spectral intensity with respect to wavenumber, vector normalization using amide I as the maximum absorbance equal to unity and mean center. Spectral expansion emphasized the most diagnostic "mid-IR" region between 650 and 1700 cm^−1 ^and eliminated a large spectral range (1800 - 2800 cm^−1^), which contains no vibrational spectroscopic information.

### Data analysis

Collection of the spectra was performed using OMNIC 7.3 (Thermo Electron Corporation). Analysis of spectra was carried out using the PLS toolbox. PLS is a multivariate method to analyze noisy, strongly collinear (correlated) data with numerous X-variables [54,55]. iPLS assisted in the selection of the most informative spectral regions. OMNIC 7.3 was used to find the absorbance values of the eighteen characteristic peaks for the different infection titers for 8 h.p.i. Differences among peak heights were analyzed using JMP version 8.0 with a one-way ANOVA. Pair-wise comparisons of peak height were performed using a Tukey-Kramer HSD test that corrects for multiple comparisons.

The analysis utilizes a "leave-one-out". In brief, the entire data set serves as the calibration, save for one sample that is employed as the prediction or validation sample. The ability to predict the concentration in this one sample is evaluated and tabulated then the one sample is returned to the calibration set, another sample is selected, and the process is repeated. In this way each sample serves as a blind assessment of prediction capability. The method the review suggests would only evaluate a small number of samples and so would not be as robust of an assessment.

### Microscopy of cell structure on crystal

An Actin Cytoskeleton and Focal Adhesion Staining Kit (Millipore) was used to visualize the architecture of cells attached to titanium oxide (data not shown) and ZnSe crystals and to perform immunohistochemistry to observe the focal adhesion points and actin filaments in the cells. Actin was stained using TRITC conjugated Phalloidin and nuclei with DAPI. The average cell height on titanium oxide was determined using a z-axis stack of cell images.

### Cytopathic effect assay

A CPE assay was conducted as described previously [3]. Briefly, a poliovirus sample of 10^7 ^PFU/ml was diluted in series. 1 ml of 1% (wt/vol) carboxymethylcellulose in maintenance medium (with 2% FBS) was overlaid onto an infected-cell monolayer. After 48 h at 37°C, plaques were stained with 0.8% (wt/vol) crystal violet in 3.7% (vol/vol) formaldehyde.

## List of abbreviations

(CPE): cytopathic effect; (FTIR): Fourier transform infrared; (BGMK): buffalo green monkey kidney; (PFU): plaque forming units; (DMEM) Dulbecco's Modified Eagle Medium; (NCS): new calf serum; (PCR): polymerase chain reaction; (RNA): ribonucleic acid; (DNA): deoxyribonucleic acid; (RMSECV): root mean square error of cross validation; (RMSEC): root mean square error of calibration; (PV1): poliovirus 1; (FBS): fetal bovine serum; (iPLS): Interval Partial Least Square; multiplicity of infection: (m.o.i.); hours post-infection: (h.p.i.).

## Competing interests

The authors declare that they have no competing interests.

## Authors' contributions

FLM conducted the experiments, performed the data analysis, participated in the design and coordination of experiments and drafted the manuscript. MR and KR conceived of the study and participated in its design and coordination and helped to draft the manuscript. All authors read and approved the final manuscript.

## Supplementary Material

Additional file 1 **Mean Absorbance Spectra of Uninfected BGMK Cells Incubated for 8 h**. Mean absorbance of uninfected cells for the spectral region between 3600 - 650 cm**^-1 ^**after 8 h of incubation on ZnSe crystals. Blue dashed lines show standard deviation. Click here for file

Additional file 2 **Mean Absorbance Spectra of Uninfected BGMK Cells Incubated for 12 h**. Mean absorbance of uninfected cells for the spectral region between 3600 - 650 cm**^-1 ^**after 12 h of incubation on ZnSe crystals. Blue dashed lines show standard deviation. Click here for file
