# Supplementary figures and images for: Detection and quantification of poliovirus infection using FTIR spectroscopy and cell culture
Source: J Biol Eng. 2011 Dec 5;5:16. doi: 10.1186/1754-1611-5-16 (PMC3260089; doi:10.1186/1754-1611-5-16)

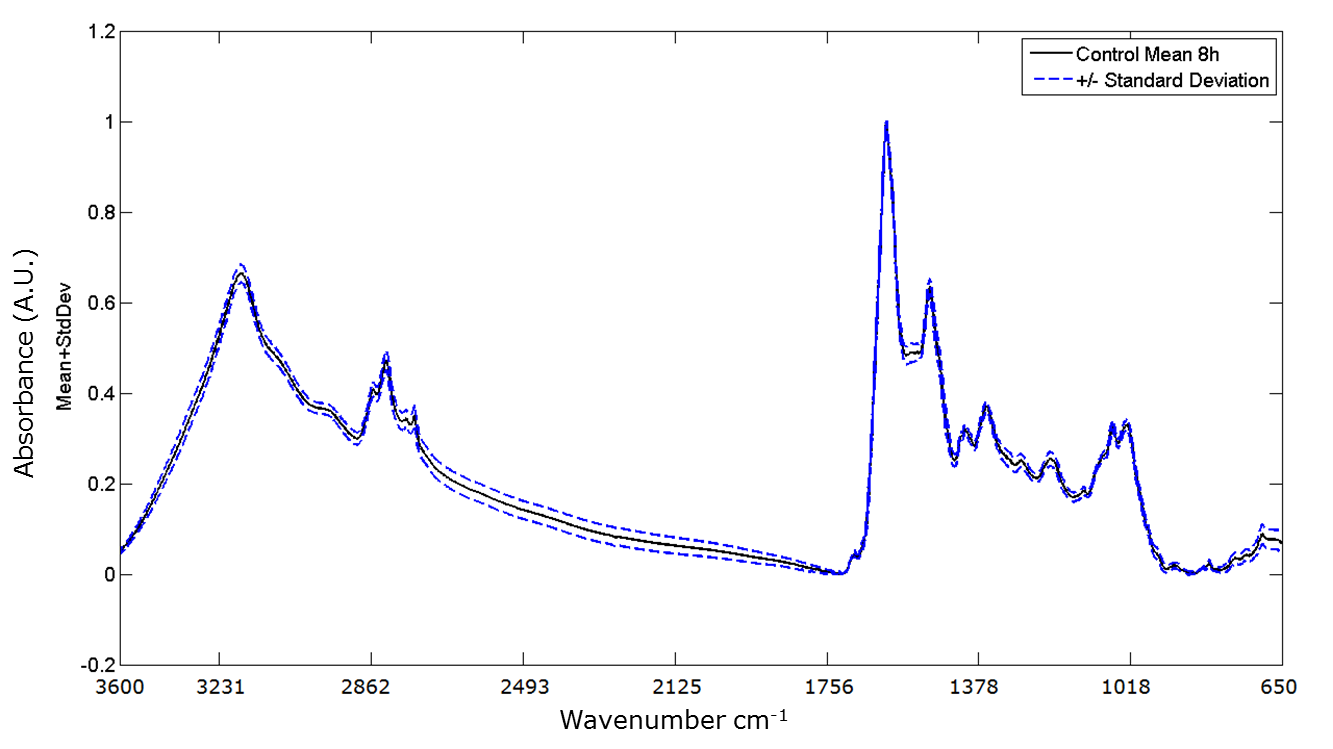

Supplement: Additional file 1 — Mean Absorbance Spectra of Uninfected BGMK Cells Incubated for 8 h. Mean absorbance of uninfected cells for the spectral region between 3600 - 650 cm-1 after 8 h of incubation on ZnSe crystals. Blue dashed lines show standard deviation. [file 1754-1611-5-16-S1.TIFF]

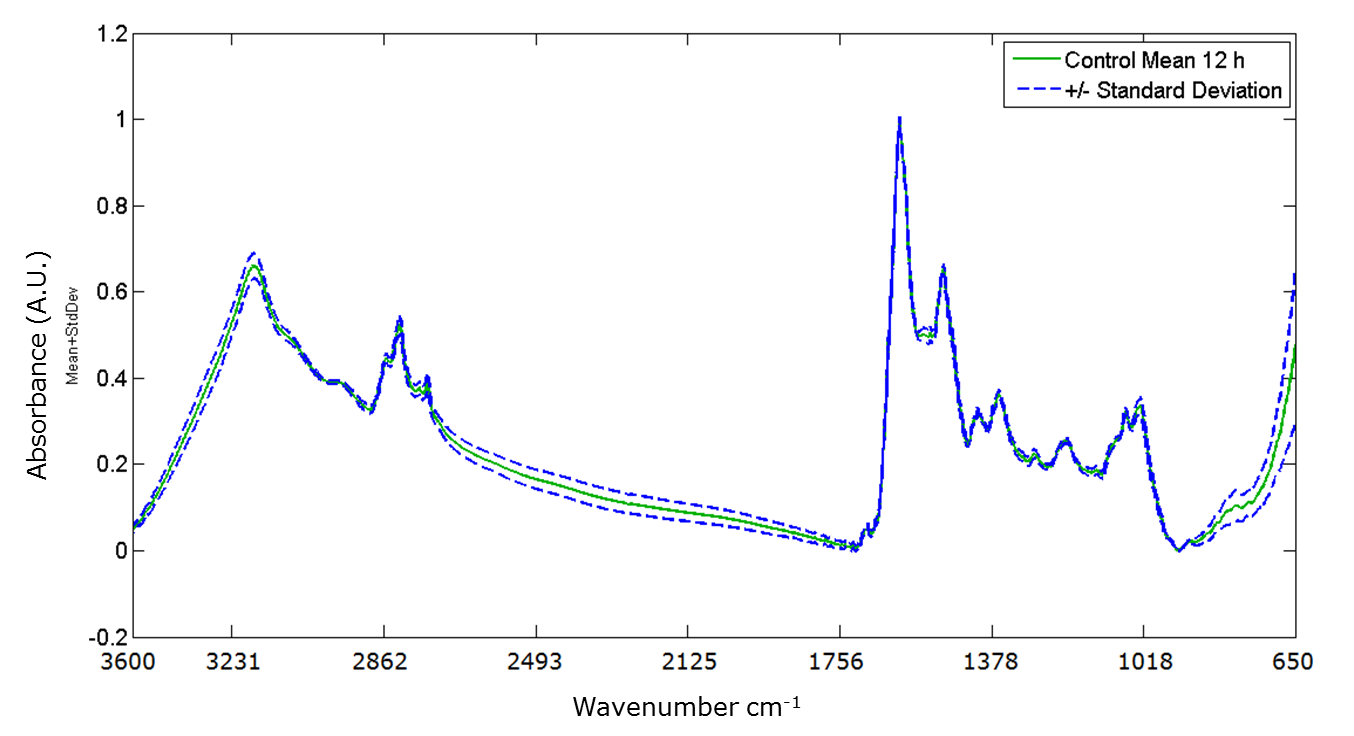

Supplement: Additional file 2 — Mean Absorbance Spectra of Uninfected BGMK Cells Incubated for 12 h. Mean absorbance of uninfected cells for the spectral region between 3600 - 650 cm-1 after 12 h of incubation on ZnSe crystals. Blue dashed lines show standard deviation. [file 1754-1611-5-16-S2.TIFF]
